# Supplementary material for: Patterns of Intron Gain and Loss in Fungi
Source: PLoS Biol. 2004 Nov 30;2(12):e422. doi: 10.1371/journal.pbio.0020422 (PMC532390; doi:10.1371/journal.pbio.0020422)
Supplement: Table S1 — Also available at http://genes.mit.edu/NielsenEtAl/. (4.3 MB ZIP). [file pbio.0020422.st001.zip › NielsenEtAl/html/805.html]

AN5579.1.NCU05837.1.MG06537.1.FG10530.1


```
 CLUSTAL W (1.82) Multiple Sequence Alignments - Introns Inserted


Sequence 1: NCU05837.1	3209 aa
Sequence 2: MG06537.1	3223 aa
Sequence 3: FG10530.1	3213 aa
Sequence 4: AN5579.1	3169 aa
Alignment Length: 3264 aa
Number Identitical Residues: 1467 aa
Alignment Score (without introns) 77683


MG06537.1 	MLEGLVAGLINRFLGMYVQNFDPAQLKVGIWSGDVNLKNLQLRREALDQLKLPLNVVEGH
NCU05837.1	MLEGLVAGLLNRFLGMYVKNFDPTQLKVGIWSGDVKLRNLELRREALDQLKLPINVVEGH
FG10530.1 	MLEGLVAGLLNRFLGMYVKNFDPAQLKVGIWSGDVKLRNLELRREALDQLKLPINVMEGH
AN5579.1  	MLEGLVANLLNRFLGIYVKNFDAKQLNIGIWSGDVKLRNLELRREALDQLHLPLNVVEGH
          	*******.*:*****:**:***. **::*******:*:**:*********:**:**:***

MG06537.1 	LGNLTLVIPWSNLRGAPVKVYIEDVFLLASPKEEAEYNEEEEERRRQRIKMDKLDSAELL
NCU05837.1	LGELTLTIPWSNLRGAPVKVYIQDVYLLASPKEEAEYNEEEEERRKQRIKMEKLDSAELL
FG10530.1 	LGELTLVIPWSNLRGAPVKVFIQDVFLLASPKEEAEYDQDEEDRRKQRLKMEKLDSAELL
AN5579.1  	VGELTLSIPWSNLRGKPVKVDIEDVFLLAAPKEDIDYDPEEEEKRANAIKMDKIESAEIL
          	:*:*** ******** **** *:**:***:***: :*: :**::* : :**:*::***:*

MG06537.1 	KERSQEGLSQEEQKRSQSFMESFATKIVDNLQVTVKNIHVRYEDSISAPGHPFALGVTLK
NCU05837.1	KERNQEGLSPEEQKRSQSFASSLVTKIVDNLQVTIKNIHVRYEDAISAPGHPFALGLTLE
FG10530.1 	KERNQEGLSQEEQKKSQSFTSSLVTKIVDNVQVTVKNIHIRYEDSISAPGHPFALGVTLE
AN5579.1  	KERNSEGMSQEEQRRNQSFTQSLVTAVIDNLQISIKNVHFRYEDSIASPGHPFAVGFTLK
          	***..**:* ***::.*** .*:.* ::**:*:::**:*.****:*::******:*.**:

MG06537.1 	EFSAVSTDGEWQPAFISDSTGTTHKLATLGALAVYWNTDATLLGTGREATTPGEEA-PTH
NCU05837.1	EFSAVSTDGEWHPTFIHDSTKTTHKLATLDALAVYWDTDAILLGPGREAIPPGHEP-KPH
FG10530.1 	EFSAVSTDGQWKPVFIQDSNTVTHKLATLGALAVYWNTDSDLLGTGREASASSEDV-MSH
AN5579.1  	ELSAVSTDAEWRPTFIQSTSGTTHKMAILGALSVYWNTDAELFGTGRGSDVGAEAQGIDH
          	*:******.:*:*.** .:. .***:* *.**:***:**: *:*.** :   ..  .  *

MG06537.1 	QEIADKFRALIPEDDEKNQTHHQFILKPVSGQAKIELDKSGKTTVPKFKANLLFDEIGLV
NCU05837.1	DVILAAFGSMIAKTNGDLPENHQFILKPVSGQAKLELDKSGSATVPKFKANLLFDEIGVV
FG10530.1 	DEMVAKFREMIGKDAQKN-SNHQFILKPVNGQAKIELDKSGDIMVPKFKANLLFEEIGVV
AN5579.1  	AELLERLRSGIDNE-----DGNQFILRPVSGRAGLEMDKTGNHEQPAVNARLLFDEIGFV
          	  :   :   * :        :****:**.*:* :*:**:*.   * .:*.***:***.*

MG06537.1 	LDDDQYRDGLMMVDLFHYFIRHQEYKKYQPKGVTPKEDPRAWFKFAGDAVLAKIHERNRR
NCU05837.1	LDDYQYRDALMMVDLFHYFIRHQEYKKYQPKGVRPKEDPRAWLQFAVNAVRSKIHERNRR
FG10530.1 	LDDDQYRDALMMVDLFHYFIRHQEYKRLQPKGVRPKEDPRAWLEFAGNAVLSKIHERNRK
AN5579.1  	LDDEQYRDALMLVDLFHCFIRHQEYRKLQPK-SRPKEDPRAWFRYAGEAVLSKIHDRNRR
          	*** ****.**:***** *******:: ***   ********:.:* :** :***:***:

MG06537.1 	WSWEYFKERRDDRLRYIELFKKRKQNPNLDPDDHDEITKLEWKLDYEDLRFWRSLARNQL
NCU05837.1	WSWDFFRERRDDRKRYIELFKKRKQDQQLSPEENDDLTKLEWKLDYEDLRFWRSLARNQL
FG10530.1 	WSWDYFRERRDDRKRYIELFKKRKQGQQMSSDETDAINSLEWKLTYEDLRFWRSLARNQL
AN5579.1  	WTWDYIKERRDDRIAYIDLFKKKKREEMLSAEETEEFNRLERKLSYEDIRFWRSLARNQL
          	*:*::::******  **:****:*:   :..:: : :. ** ** ***:***********

MG06537.1 	KKENAAALRNQPK-KAEPQQQGWLSWVWGAKPEAEHQPAD--EDENTKITEEQRKELYEV
NCU05837.1	KRENAEALRNRPPPSAQPQQQGWLAWAFGSKPSQQQQQEKQLEDENTQITEEQRKELYEV
FG10530.1 	KKENAEALKNQPQ--QPQQQQGWISWVWGSKPQETIEHNE----ENTQMTEEQRQELYEA
AN5579.1  	RKENVGVKK-------PPRQQTWSEWIWGSKKEDS---------EETAMTEEQRQELYNA
          	::**. . :         :** *  * :*:* .           *:* :*****:***:.

MG06537.1 	IDWDEKTALAESIDEPRETVKLQIEASLSTGSLTLKQNPHGDSRDLVSLHFDLFKAKALK
NCU05837.1	IDWDEKTALAAEVDVPRDTVEMQLEASLSTGSFTLKQNPHGDSRDLISLHFDVFKAKGLK
FG10530.1 	IDWDEKNAIAEEVDMPREAIKMCIETSLSTGSFTLKKSPHDNAADLLSLHFDVFKAKALQ
AN5579.1  	IDWDEKKTITESVDVPREWVKLQVNAGLKAGSLTLVRGPHGDANEVMKFVFDNFRAKALQ
          	******.::: .:* **: ::: :::.*.:**:** :.**.:: :::.: ** *:**.*:

MG06537.1 	RVDSIFLDLGLGGLRVNDGTTPDTFFPEIVRVKD---APNNKRRKRLSIVELEEHS----
NCU05837.1	RPDSFLADVSLGGLRVNDGTTPNSLFKEIVRVKD---APDTHLAPRLSIAELEQSS----
FG10530.1 	RKDSMLANVSLGGLRVNDGTTPDSVYPEIVRVKD---APTNNQRKRLSLAELEQPE----
AN5579.1  	RPDSFFLDLDLGGLRVYDGTTEGSLYPQIVRVKDSLPEPKHRLSQISSSGEFDPEEGVDD
          	* **:: ::.****** **** .:.: :******: . *  .     *  *::  .. ..

MG06537.1 	---EEAFFRLQVEQNPLDAQGDIAVTAKLNPLEIVWNPNIVVGVVDFFRPPERHMESITA
NCU05837.1	---DEAFFQFQVEQNPLDGQGDIAVTAKLKPLEIVWNPNVVVGVVDFFRPPERHMDSINA
FG10530.1 	---EDPFFQFEFEQNPIEREGDIAIVGKMKPLEVIWNPNFVVGIADFFRPPERHMESITA
AN5579.1  	LEDEDSLFHLQLEQNPLESDADSVVKVKLKSIEVIYNPKFIVEITRFFRPPERHMESIGA
          	 ..::.:*:::.****:: :.* .:  *::.:*:::**:.:* :. *********:** *

MG06537.1 	LMESAGATVEGLREQTRAGLEFALEEHKTINAKLDLQAPLIIIPQSITQAKSTCVILDAG
NCU05837.1	LMETAGATVEGLRAQTRAGLEFALEEHKTLNAKLDLQAPLIIIPESITSKQSTCLILDAG
FG10530.1 	LMESAGATVESIREQTRAGLEFALEEHKTINAELDLQAPLIIVPVSITTENSTCLIVDAG
AN5579.1  	ILDSAGATVEGIRQQTRAGLEFALQEHKKVDAQFDVHAPLIIVPESITHESSLCLIIDAG
          	::::******.:* **********:***.::*::*::*****:* ***  .* *:*:***

MG06537.1 	HISVNSELVDKESMKQVQSKQSQTYTDEDYKRLESLMYDKFIVKLTSTQLLIGPSIDETK
NCU05837.1	HISVNSELVDKDTMKEVQSKQRQSFTEEDLKRLESLMYDRFLVKLSSTQVLIGPSIEETK
FG10530.1 	HIHVNSELVDQGTMKEIQSKQKQTYSDEDLKKLESVMYDKFIVKLTSTQVLIGPSVDETK
AN5579.1  	HASVNSELVDRQTMKDLQSKQKRQYDTGDYKELEHLLYDRFLIKLDSTQVVIGPGIETAK
          	*  *******: :**::**** : :   * *.** ::**:*::** ***::***.:: :*

MG06537.1 	TQLMERDESKMMHVVDKINVDFVVAISILPKAPNLTRMKVSGHMPVLHAVASDTKYKTLM
NCU05837.1	QQLVDKDD-KMLHIVDKITVDFVVETSILPKAPNLTKLRVSGHLPVLHASVSDAKYKSLM
FG10530.1 	AQLVEKSDEAHLHVVEQINVDFVVETSILPKAPNLTKFKVSGHLPMLHATVSDTKYKNLM
AN5579.1  	AQLGPDVESKNLHIIDRINVDFVLEMCIVPKLTELTRTRISGHLPELHASMSDTKYKGLM
          	 **    :.  :*::::*.****:  .*:** .:**: ::***:* ***  **:*** **

MG06537.1 	QIINVAIPKLGNEAESTMPVEDKPQPKRPRLSSGVSSRSGVRKSHHRKSSQFINFAPQQQ
NCU05837.1	RIIDVAIPKLGGADTPSQPAGQ--PGLRPRLASNASTAS-HRLLEHRPSAQLLPFSTSHQ
FG10530.1 	KVIDVAIPKFG--GTSAERIEQHREASRPRLKSNSSNRSRRKSQRERRQSTPFPFMQS-E
AN5579.1  	KLIDIAIPRFDDEAQDTN---------------TSKNKEGTSITHTRARSSSFQPIGQRE
          	::*::***::..    :                  .. .     . *  :  :    . :

MG06537.1 	TAIVLDDSIDDEDE--FEDASEGSTKQQLKMQQRTFEFKFAVDELRGSLYRSDPERKKPD
NCU05837.1	QAIILDDDDHDEDEDAFEDAKDGSATDQLKLQQRNFEFKFAVDELKGSLYRSDPDGKAPD
FG10530.1 	TAVVLDDMDEDDDD--FEDATDGGDSEQLKVQQRIFEFKFKVDTLKGSLYRSDPDGRKPD
AN5579.1  	LPVVDEESDIEDSD-----QISKSVDKLPDIHRRDFEFKFTVGRLRGSLFRSDSTDPFRE
          	 .:: ::   ::.:       . .  .  .:::* ***** *. *:***:***.     :

MG06537.1 	ALLVELVAQNFDLEFFIRQFDMVAEVSLGSVTVDDFVD-NPPAEFKSIVSSGDIEDRKQK
NCU05837.1	QLLVDLVAQRFDLQFYIRPFDMSAEVSLGSVTVDDFVD-NPPAEFKAIVSSGDVEESGEV
FG10530.1 	ALLVELVAERFDLEFYTRPFDMVADVSLGSVTVDDFVD-NPSAEFKSIISSGDSEDLKEG
AN5579.1  	QLLVELVAEGFALDFYMRPFDMVAEVILKSLSVDDYIEANPVPEFKKIISSTG-FDADEN
          	 ***:***: * *:*: * *** *:* * *::***::::** .*** *:** .  :  : 

MG06537.1 	RELVHVKYVKVKKESPEFMSVYDGVETNVNAAISTINLIVTRKTLLTLLDFILITFAPGN
NCU05837.1	QDLVRVKFVKVRKDSPEFMPVYDGVETNLDVAISTINLVVTRKTLLTLLDFVLVTFTNTA
FG10530.1 	RSLVHVKYIKVNPASPEFMSVYEGVETNINAVVSTINLIVTRKTLLTLLDFILITFTNNN
AN5579.1  	KDLFYLRFVRVKPESPEFHSTYEGVAMNLDISVSTINLVVTRKTLLTLLDFILLTFTNPQ
          	:.*. :::::*.  **** ..*:**  *::  :*****:************:*:**:   

MG06537.1 	NDNNANTQQKAISPDGSE----VDIVVDPPPGQQPQQ-GGAIRVKVDLKSIRMILNNDGI
NCU05837.1	NPNPP--PPNAITDDGSE----TNIEVVPPAPPQQIS-DSAIRVKVDLKSIRLILNNDGI
FG10530.1 	NDNNQPNNNKAILDDDDEDCESINTAVAVANEPQSTSSNGSIRVKVDLKSIRLILNNDGI
AN5579.1  	QPNQD--TQAALTIEDTES-----------AEQQPQQ-EGKIRIKADLKSIALILNNDGV
          	: *       *:  :. *.              *  .  . **:*.***** :******:

MG06537.1 	RLATLSFNQADVGVFLLGKTMRVSTKLGDLSLVDDVNQGVSEESSLRKLVTIQGDDLADF
NCU05837.1	RLATLSFNHADVGVFILGKTMRVAARLGDLNLVDDVNLGVSEDSSIRQLVAIQGEELADF
FG10530.1 	RLATLSFNKADAGIFLRGNTMRISAKLGDLSLVDDVNLGVSEDSHLRQLVTIQGDDLADF
AN5579.1  	RLATLSLHTADVGIFLVGRTMLIQSRIGSLTLIDDVNTGAKKSSDLRRLLTIEGDNFADF
          	******:: **.*:*: *.** : :::*.*.*:**** *..:.* :*:*::*:*:::***

MG06537.1 	RYETFDAE-SKSYPGHDSFIYLRAGSVKINFMEEPFRKIIDFLVKFGKMQAIFNAARQAA
NCU05837.1	RYETFDSGRIESYPGYDSSIYLRAGSVKINFLEEPFRKIVEFLVKFGKMQAIYNAARQAA
FG10530.1 	RYETFDATNPKAYPGYDSSVFLRAGSVKVNFVEEPFRKIVDFLVKFGKMQALYNAARQAA
AN5579.1  	KYETFDPE-APEYPGYDSEVFLRSGSIKINFLEEPYRKIINFLVKFGKMQAIFNAARRAA
          	:*****.     ***:** ::**:**:*:**:***:***::**********::****:**

MG06537.1 	ANQANQLQQGNSRIKFDVVVKTPIVVFPRVVLPSQPKRDLMTAYLGEIYAQNKFAPLDDS
NCU05837.1	ANQANQFQQSPSKVKFDVVVKTPIVVFPRHVRPDRPKRDLITAYLGEIYAQNKFVPLDDS
FG10530.1 	MNQANQLQQSPSRFKFDVVVNTPIVVFPRVQKPGQTERDVVTAYLGEIYAQNKFTPLDDS
AN5579.1  	ANQANQLQENASRMRFDVVVMTPILVFPGAMSEDRP-YDTVTAHLGEIYAKNTFVPLDEE
          	 *****:*:. *:.:***** ***:***     .:.  * :**:******:*.*.***:.

MG06537.1 	E-NSEVVQKLSVGIRNIRLTSLFHYPDDVSEELELIDHVDLGFKMTYAEHKEGAKRPDLE
NCU05837.1	E-NAEIAMKLTAGIRNIKLTSNFHYNDGQEEELELIDKVDLGFQITYAEHKEGVKRPDLE
FG10530.1 	E-DSEIAMKLSAGIRNIKLTSDFHYPGGVSEELEMIDHVDLGFNITYAEHRHGAKRPETE
AN5579.1  	QPDSPAVNLISTGIRNIRLTSRFHYAGGAVEELEMIQKVNLDFSICYLEHQPGNPRPDME
          	:.::  .  ::.*****:*** *** ..  ****:*::*:*.*.: * **: *  **: *

MG06537.1 	IEGHMTDFNLRITQYQLKFLLEISRSVPAAFAGDPDQSVDEAVEQQVDNHTLENARSQSG
NCU05837.1	IEGNMSDFNLRLTQYQLNSLLEISRSVPAAFSGDAEQ-NRLDAERDVDDRTLSKARTMSG
FG10530.1 	IEGTMSDFNLRLTQYQVKFLLEISKSVPAAFAGEGGD-KEEEAAKAVDEGTLQRARTSNF
AN5579.1  	IEGTMSPINLRVSQKQLKFLLDITRTAPNAFVTDSEQ-QELEAKEALPSLTDSGKETESS
          	*** *: :***::* *:: **:*:::.* **  :  :     . . : . * .  .: . 

MG06537.1 	ERGSSGDNQLVDLSPELGTQAETWTRLDLVFVVHTIGLELLNGAEDRPIGDSEKASLSRF
NCU05837.1	FDDNGGNEQRIDMRPELGPKAETWTKLDLIFTVNTIGLELINAPEDRPVGDLDAASLSKF
FG10530.1 	EDKSGDEKKLVDLGPELGSLGQEWTKLDLVFRVNTIGLELINAEENEPVEDIAKSSLSRF
AN5579.1  	LVQSTNQHNVATTDPNG--KGENWVRLDMIFKADSVGLELILGKDDAPIGSLDDASLSKF
          	   . .:.:     *:    .: *.:**::* ..::****: . :: *: .   :***:*

MG06537.1 	SLDDSKLKTRIQSNNALEAEFVIHSFTIHDSRPGETNKFRKIMTSRNKNV-QQFMASVSM
NCU05837.1	SLDSSRLKTRMDSNGSLEAEFVIQSFTIYDTRQRETNKFRRIMTSGNKDV-QQLMASVTM
FG10530.1 	SLDDTKVKTRMLSDSSLEAELLIRSFTIYDSRPRDTNKFRRIMSSMNKDV-QQLMASVTI
AN5579.1  	SLNDTQVKLRMLIDGSLESELLIHSFSIRDSRKQETNKFRKIMSLINNDVKQQFMASVSI
          	**:.:::* *:  :.:**:*::*:**:* *:*  :*****:**:  *::*.**:****::

MG06537.1 	TGGDKRDVIALVAIDNPRVIFALDYLFTIQRFVTTALTVEEPHVIEELQSPIETPDES-D
NCU05837.1	SGGKERNIIAMVAVDSPRVIFALDYLFAIQNWVTVGLKVPEPEVPEK--SPLDTPDDMSD
FG10530.1 	SGGKEKSLIAMATMDSPRVIFALDYLFSIQKFAIEALAVDDSSPMDDESMADNTPDES-D
AN5579.1  	SPGPAKHLIAMLTIDSPRIILALDYLSALQSFANSVFSADEPQEDEE---DIESPEEDEP
          	: *  : :**: ::*.**:*:***** ::* :.   : . :.   :.     ::*:: . 

MG06537.1 	AESMQVTFSG---RGSARPSTSAEAG-QAAKGPENTAPMNLAFRVNVVDAQVILIANPLS
NCU05837.1	ADSVAVSYTG---RRSESIDQRLGPSELQTEPPKQESKTRLAYRVNVVDAQIILIANPLS
FG10530.1 	AESMQVTLAGGASRPRSELSRRQSEDVAKQGDNKEESSMSIAFRVNLVDAQVILIANPLT
AN5579.1  	RSSMAESTDG------------SIRSPSLEEEPQAPSQMTVSFRVSLVDAQIITIANPAI
          	 .*:  :  *               .       :  :   :::**.:****:* ****  

MG06537.1 	ASSEAIVLGTKQVLLSKQHALTLQVSEIGMFLCRMDKFEESRLRIIDDFSIRTSMDTSIE
NCU05837.1	SSSEAIVLGTKQVLLSQQHALTFQVSECGMFLCRMDRFDDARLRIIDDFSIQMSMDTTKP
FG10530.1 	SSSEAVVLSIRQMLLSKQHALTFQISEIGMFLCRMDKFETSRLRIIDDFSVQLSMDGSKP
AN5579.1  	PHTEAIVLGAKEVLFSKQNVSTLHVSKIGMFLCRMDKFETSRLRILDDFTLELSMDSRSQ
          	. :**:**. :::*:*:*:. *:::*: ********:*: :****:***::. ***    

MG06537.1 	----NQTNIHIDIEPLILRLALRDILVALQIIAKASELSGNDE-PTPKTGTADAKAKELR
NCU05837.1	----STTNIHVDIEPLILRVSLRDILLVMQIVSKASELSGNTP-TEPKGTTSDQKAKQLK
FG10530.1 	----QATDINVDVEPLVLRLSLRDILLVLQIVSKASELSGNEPKQQVKESPADQKARELS
AN5579.1  	DRGASLTSIDIHVEPLVLRLSLRDILMAIQIVNKASEMR-AARILPAETGETKALPESTG
          	. .:. *.*.:.:***:**::*****:.:**: ****:         :   :.  ...  

MG06537.1 	QAGLKQRTASGKGRSTVAPNKTKSATVAPSTRSQQGPRSGGVHDIAKPPIMREELTATIE
NCU05837.1	SSDMKQRTASGKGTSTVANKSKRTGTKTGTTDPRSQKAGQQPHDITKAPPRYEQLTAAIE
FG10530.1 	NAGIKQRSASGRGQSTIGGRSKVTATSHAVTHGADTRGGKAPEKVAQQN--YETLSATVE
AN5579.1  	TFGSRTRRKSSVGKQASAAATKRSHRRSIGAVNAIDRKPVNKRSVVLQR---EELKAQVD
          	  . : *  *. * .: .  .. :      :           ..:.      * *.* ::

MG06537.1 	GIRVVLIGDLHELPIMDLSIKDFTTSAEDWSSNLKAETAIEMYINVYNFSKSAWEPLLEP
NCU05837.1	GIRLVLIGDVHELPILDMSIKSFTTSAENWSSNLKAEAAIEMYTNIFNFAKSAWEPLIEP
FG10530.1 	GIRVVLIGDVHELPILDIGIKNFKASAENWSSSLKAETAIDLYSNVYNFAKSSWEPLLEP
AN5579.1  	GVRVILIGDLHDLPLLDWSVKKFNVDARDWSSTLTADTSFDTFVNVYNFSKSAWEPLIEP
          	*:*::****:*:**::* .:*.*...*.:***.*.*::::: : *::**:**:****:**

MG06537.1 	WKVGFGVAKNPESGLLSIDVTSKQIFDVTITTSTIALASKSIEYLRRDEDVLGKPRGVEA
NCU05837.1	WQVGFGIARDQSTGLMSVDVSSKKTFDVTLSTASIALLSKSMAFLAQDEDVLGKPRGVEA
FG10530.1 	WQVGFGVAKDPVSGLMSIDVASKKVFDVTITTASIALASKSFQFLTSDEDVLDKPRGVEA
AN5579.1  	WQLGIHMAKEVNPDVFSVDVYSHKTMELTVTSATIDLASKSFQFFYTDEDVLSKPRGADA
          	*::*: :*::  ..::*:** *:: :::*:::::* * ***: ::  *****.****.:*

MG06537.1 	PYRLRNYTGFDVVVTAKNPTSNENITLRLEEGQEAPWSFEDWEKMRESLITESS-SSSVA
NCU05837.1	PYRIRNYTGFNIIMQAKREGTEETTPLRLEDGQEVPWSFENWEKVRESLSGESHGTSNVA
FG10530.1 	PYRIRNYTGFDVLVHSKSPTSEEPINLRLEDGQEAPWSFEHWEKMRENLLTESN-QNNVS
AN5579.1  	PYRIRNYTGFDLHVWADVAEGEDGPAAKLADGEEYPWRFEDSTSMRETLAPEGQ-AGLVG
          	***:******:: : :.    ::    :* :*:* ** **.  .:**.*  *.   . *.

MG06537.1 	VQLEGSGFDPVKNIRLNREGEFLYGLKPKADKVLHRMLVEVSLGNDNVKYVTLRSPLLVE
NCU05837.1	IQLEGSGFDTVKNIRFTREGEFLYALKPKTDEVLHKLMVDVKLGTDNVKYVTLRSPLLVE
FG10530.1 	ISLEGSGFDPVKNVRLNREGEYLYGLRPKADDVLHRLCVDVELGTDNIKYVTFRSPLHVE
AN5579.1  	VKLEGSGFDSVTRIPVVREGEFLYNLKPKKDTVLHRLLVEVKLGPDNVKYITIRSPLLVE
          	:.*******.*..: . ****:** *:** * ***:: *:*.** **:**:*:**** **

MG06537.1 	NQTQIPVELGVYDAQDGHLLKIEKIAPGEARPAPVGAVFLKSLLVRPDSGFGYQWSSETL
NCU05837.1	NETDIPVELGVYDAQEGHLLKIEKIAPGESRPAPVGAVYTKSLLVRPDPGFGYGWSNDTL
FG10530.1 	NATEIPVELGIYDAQDGHLLKIEKINPGDSRPAPVGAVFEKRVLIRPDSGFGYQWSNDQL
AN5579.1  	NNTQIPVELGVFNPQDGHLLKIEKILPGDARPAPVGAAFMHSIVIRPDQGFGYDWSHEQL
          	* *:******:::.*:********* **::*******.: : :::*** **** ** : *

MG06537.1 	WWKDLLKRPTRTMVCKGE---QGEPFYFQVHASYDKTNPLTS2VYPYMRVKLSAPVVLEN
NCU05837.1	WWRDLLKRPTKTLMCKGE---HGEPFYFQLSARYDKANPATR2TYPYMRLKLSAPVTIEN
FG10530.1 	YWRDLLKRPTKQVVCKGE---NGDPFYFQVHARFDKTNPLTK2HYPYMKIKLSAPVTLEN
AN5579.1  	YWKDLMRRPTRTIKCLSEGAQQSPPFYFQTNATFNARDPLTK~IYPYMRIRIFAPVEIQN
          	:*:**::***: : * .*.:.:. *****  * ::  :* *   ****:::: *** ::*

MG06537.1 	LLPYDFKYRIYDKNTKKDWTNFLRKGGVSPVHVVELSHLLLLSIDMQDTVFKPSDFAIIN
NCU05837.1	LLPYDFKYRIYDKNTKKDWSNFLRKGGVSPVHVVELSHLLLLSIDMQDTVFKPSEFAIIN
FG10530.1 	LLPYDFKYRIYDKATKKDWTNFLRKGGVSPVHVVELSHLLLLSIDMQDTVFKASDFSIIN
AN5579.1  	LLPYDFKYRIYDKNTRKDWTNFLRKGGVSPVHVVELAHLLLLSVDLEDTVFKQSDFSIIN
          	************* *:***:****************:******:*::***** *:*:***

MG06537.1 	SGQSEEYKKEMALTCRDEQGLVLNLKMHYYRIPNGGGAFKVTVYSPYVILNKTGLDINIK
NCU05837.1	SGTSEDFRKETNLVCKDDSGLALNLKLHYFRVPDSGGAFKVTIYCPYVILNKTGLDVSIR
FG10530.1 	PGNNEEFRKEGRIVVKDDQGLPLHLSLHYFKIPNSGGAFKVTVYSPYVVLNKTGVDVRVR
AN5579.1  	-GNAHDFRREHTLSLKDERGLPLKLKLHYFNVPDSGGAFRVSVYSPYLILNRTGLTMEVQ
          	 *  .::::*  :  :*: ** *:*.:**:.:*:.****:*::*.**::**:**: : ::

MG06537.1 	SKGFMQHAKAAAGGPGQGRQSLIDVSDNERLKALPIMFSFANDDQRNRAMLKVGDSEWSK
NCU05837.1	SKGFMQHAKAAAG------QQLVDVGESQR-KAQPIMFSFGSDDHRNRALLKVADSDWSR
FG10530.1 	AKSFLQQAKPAAG-----QFPLMDTSNQDHPKALPFMFSFGNDDHRNRALLKIADSEWSK
AN5579.1  	SKAFLQSARSAAG------QGLRAEAGSRQRRVRPYMYSYATEDQKNRSIIRIGDSSWSK
          	:*.*:* *:.***        *   . . : :. * *:*:..:*::**:::::.**.**:

MG06537.1 	GQSFDAIGSTSDVVLQSTSRNAEIHVGISVQSGEGKYKMTKVVTLAPRFIVQNKMGEEIN
NCU05837.1	PQSFDAIGSTTEVVLNSPSRNAEIHIGVTVDTGHGKYKMVKTVTLAPRYVIQNRLGEDIN
FG10530.1 	PQSFEAIGSTTEVVLNSANKDKEIHVGVTVESGQGKYKLTKVVTLAPRFVLHNKIEGEIL
AN5579.1  	PQSFEAIGSTFEVVLPDRDGRSEYHAGVSVAEGEGKYKLTKIVTIAPRFVLKNKLEEDIL
          	 ***:***** :*** . .   * * *::*  *.****:.* **:***::::*::  :* 

MG06537.1 	IREPSSSSLMSLQKDALQPLHFLQRTSVKQLCLCYSGVNNQWSAPFNISDIGTQHVKIAR
NCU05837.1	IREPSSSSVLELKTGALRPLHFLQKGAVKQLCLCYPGVDNQWTAPFNISDLGTTHVKIAR
FG10530.1 	VRESSSSGYLTLSDGALQPLHFMQKSKVKQLCLCYPGVNNQWTSPFNIADIGTQYVKIAK
AN5579.1  	VREPGSSNVTDIKGGDLVPLHFLRQAGEKQLCLCFPGVNNQWSSPFNIADVGTVYVKLAR
          	:**..**.   :. . * ****:::   ******:.**:***::****:*:** :**:*:

MG06537.1 	TGHRQQLVRAEVLMEDATIFVTLSKETKNWPFSMRNESDTEFTFYQA~NPNMDEDGAEDR
NCU05837.1	AGQRQRLVRVETLMEESTIFLNLTMETKNWPFSMRNESDTEFTFYQA~NPNLDEDGIEDH
FG10530.1 	AGQRQNLVKVEILMENSTVFVNFSMETKTWPYSVRNESDIEFMFWQA~NPNIDEEGVEDR
AN5579.1  	QNQRQRLIKIDVIMEAATLFVHLSLEHRNWPFSMRNESDVDFMFYQT0NPNLEDDEEDRT
          	 .:**.*:: : :** :*:*: :: * :.**:*:***** :* *:*: ***::::  :  

MG06537.1 	SGWRQIRYRLPPRSIMPYAWDFPAAKHKEVVISANGKERHVKLAEIGNLVPMKFVAAS--
NCU05837.1	SGWRPVRYRLPPRSIMPYAWDFPAAKNKEICISANGKNRHVKLTEIGNLVPMKFVDAN--
FG10530.1 	SGWRQIRYRLPPRSVMPYAWDFPAAKFREIIISANGKERHVKLAEIGNQIPMKFVTAS--
AN5579.1  	NTWRPIRYRLPPRSIMPYAWDFPATKNKSLVLACKGKERRIRLTEIGNLIPMRIPPSQPG
          	. ** :********:*********:* :.: ::.:**:*:::*:**** :**::  :...

MG06537.1 	GQPKIIDINVAADGPTQSLILSNFKQSKSLYRQKSHLHRSNTASSSTTDAGFEVKSQDLG
NCU05837.1	GQTKVIDINVIADGPTQTLILSNFKQSKSLYKQK-------SNASSTDRSTFEAKELDTG
FG10530.1 	GNQKIVDINVAADGPKQTLILSNFRASKSMYKPK------ALSRTNTGLEAFEVKNQDTG
AN5579.1  	EAQKIIDINIVADGPTQTLVLSNFKASKSIYRQQR-----SQTSQTSINTGFEVKEFDSD
          	   *::***: ****.*:*:****: ***:*: :           .:    **.*. * .

MG06537.1 	TTFQAQLKLAGIGVSLITSQLKELAYITLRDVELKYSESPLYQTVSLAVKWIQIDNQLFG
NCU05837.1	VTFRAQLRLSGIGISLINSQLKELAYITFRDVQLRYSDSQLYQTVSLAVKWIQIDNQLYG
FG10530.1 	ATFRAQLKLAGIGVSLVNAQMKELAYLTFRDVQLRYSDSPLIQTISMAIKWIQIDNQLYG
AN5579.1  	VNFKAQLHLGGIGISLINQKMKELLYMTFRDIEVKFKESKAYQTLNTTIKWIQIDNQLYG
          	..*:***:*.***:**:. ::*** *:*:**:::::.:*   **:. ::*********:*

MG06537.1 	GIFPMVLYPSVVPKRAQEIDAHPSLHAQVTRVKDDSYGVLYIKYATVLLQQMTVDLDEDF
NCU05837.1	GIFPIILYPSVVPKRAQEIEAHPSLHAMVTRVKDDSYGVEYIKYATILLQEMTVELDEDF
FG10530.1 	GIFPMILYPTVVPKKAQEVEAHPSLHFMVSRVKDDSYGVLYVKYATILLQEMSVELDEDF
AN5579.1  	GIFPMLLYPSVVPKTGKEMEAHPIFHTMITRVKDDSYGVLYIKYATLLLQQMTLELDEDF
          	****::***:**** .:*::*** :*  ::********* *:****:***:*:::*****

MG06537.1 	VYAVLDYSKVPGTVWTIEEEDGTLCDDNLDIPRPQQQQAAQDIYFELLNIQPMQLDLSFV
NCU05837.1	VYAVLEFSKVPGASWSSSDEEDKLCDDSIDVPQPKQLDSGRDIYFEVLNIQPMQLDLSFV
FG10530.1 	VFALLDFTNVPGASWTEVDEAGKLCDEDIDIPEPSGLPAGTDIYFEVLNIQPMQLNLSFM
AN5579.1  	IFAMLDFTKASGAAWAG-KHEDKLCEEQLNIPEPQTEGAAQDVYFELLHLQPMQLDISLM
          	::*:*::::..*: *:  .. ..**::.:::*.*.   :. *:***:*::*****::*::

MG06537.1 	RTERINAEDKTSSRNPLMFFLNVMTMAIGNVNDAPLRLNALMLENVRVSIPLLVQNVSNH
NCU05837.1	RTERVNVEDKTSSKNPIMFFFNVMTMAIGNINDAPVRFNALMLENMRVSVPVLIQRISNH
FG10530.1 	RTERVNAEDKASSHNPIMFFLNVMTMAVGNVNEAPLKFNALILDNVRVTTAVLIQNFSSH
AN5579.1  	RTERVNVEDTLQPSNPLMFIVDVMTMSMGNVNDAPIKLNALMIENARVSFPVLASNIRRH
          	****:*.**. .. **:**:.:****::**:*:**:::***:::* **: .:* ...  *

MG06537.1 	YSQEALYQVHKILGSADFLGNPVGLFNNISSGVADVFYEPYQGLIMSDRPEDIGVGLARG
NCU05837.1	YSQEALYQVHHILGSADFLGNPVGLFNNISSGFADIFYEPYQGLIMSDRPEDFGIGLARG
FG10530.1 	YSQEVMYQIHKILGSADFLGNPVGLFNSISSGVTDVFYEPYQGLILSDKPEEFGLGIAKG
AN5579.1  	YTQEFLRQIHVVLGSADFLGNPVGLFNNVSSGVAAIFYEPYQGLVMTDRPQELGYGIAKG
          	*:** : *:* :***************.:***.: :********:::*:*:::* *:*:*

MG06537.1 	AASFAKKTVYGFSDSFSKFTGAMSKGLAAATLDKQFQDRRRITRARNRPKHALYGVTAGA
NCU05837.1	AGSFFKKSVFGFSDSFSKVTGSFAKGLAAATMDKQFQDRRRITRARNHPKHALFGVTAGA
FG10530.1 	AASFAKKTVFGFSDSFSKFTGSLSKGLAAASLDKQFQDRRRITRARNKPKHALYGVTAGA
AN5579.1  	FGSFAKNTVFGFSDSISKLTGSMSKGLAAATLDKEFQDQRRMSKTRNRPKHALYGITAGG
          	 .** *::*:*****:**.**:::******::**:***:**::::**:*****:*:***.

MG06537.1 	NSLFTSVASGVGGLARKPLEGAEQEGALGFFKGVGKGVLGFTTKPVIGVLDLASN~VSEG
NCU05837.1	NSLFTSVASGVGGLARKPLEGAEQEGALGFFKGIGKGVVGLATKPAIGVLDFASN~VSEG
FG10530.1 	NSFITSVASGVGGLARKPLEGAETEGALGFFKGVGKGFIGLATKPAVGVLDMASN~VSEG
AN5579.1  	RAFGNSIVSGIEGIARHPLHGAEKEGIQGFFKGVGKGFLGLATKPAIGAFDLASN1LAEG
          	.:: .*:.**: *:**:**.*** **  *****:***.:*::***.:*.:*:*** ::**

MG06537.1 	IRNTTTVFDGSELDRVRLTRFISKDGIVRPYSQREALGQSWLKQVENGKYFDEEYVAHLE
NCU05837.1	VRNTTTVFDGTELDRVRLPRYIPPDGIIRPYSQREALGQSWLKQIDNGKYFDEQYIAHLE
FG10530.1 	IRNTTTVFDQDGLDRTRYPRYIPQDGIVRPYNPREALGQYWLKQVDNGRYFDEQYIGHLE
AN5579.1  	VKNTTTVFDAEGLDRVRLTRFVGMDGIVRPYSQREALGQFWLKTADDGKYFNEDYIAHLE
          	::*******   ***.* .*::  ***:***. ****** ***  ::*:**:*:*:.***

MG06537.1 	LPREDVVVMVTYSRILLIRSRRLTTEWDIPLKDVQTISKERTGLSLVLRGGQNGPFIPVG
NCU05837.1	LPTEDKVVMVTFARILLIRSRKLQTEWDVPLKDVQTIAKERTGLSLTLRGGTNGPFIPIG
FG10530.1 	LPKEDMVVMITYARILLIRSRRLTSEWDVPLKDVQTIAKERTGVSLSLRGGANGPFIPIG
AN5579.1  	LPGRDMLIMLTYDRIMLVRSKKLETDWDIRLTDIQTISKERTGMSIMLKGGANGPFIPVQ
          	** .* ::*:*: **:*:**::* ::**: *.*:***:*****:*: *:** ******: 

MG06537.1 	EESGRAFLYRSVAIA~VEEFNRRFRALE
NCU05837.1	EESGRTFLYRQVAIA~VEEFNRRFRGLE
FG10530.1 	EGSERGFLYKMIGVA~VEEFNRRFRSGE
AN5579.1  	DESARNWLYKQIAIA1VNAFNEKYTNRA
          	: * * :**: :.:* *: **.::
```
